# Toxicity of Nano-Zero Valent Iron to Freshwater and Marine Organisms

**DOI:** 10.1371/journal.pone.0043983

**Published:** 2012-08-30

**Authors:** Arturo A. Keller, Kendra Garner, Robert J. Miller, Hunter S. Lenihan

**Affiliations:** University of California Center for Environmental Implications of Nanotechnology, Bren School of Environmental Science and Management, University of California Santa Barbara, Santa Barbara, California, United States of America; University of California, Merced, United States of America

## Abstract

We tested whether three commercial forms (uncoated, organic coating, and iron oxide coating) of nano zero-valent iron (nZVI) are toxic to freshwater and marine organisms, specifically three species of marine phytoplankton, one species of freshwater phytoplankton, and a freshwater zooplankton species (*Daphnia magna*), because these organisms may be exposed downstream of where nZVI is applied to remediate polluted soil. The aggregation and reactivity of the three types of nZVI varied considerably, which was reflected in their toxicity. Since levels of Fe^2+^ and Fe^3+^ increase as the nZVI react, we also evaluated their toxicity independently. All four phytoplankton species displayed decreasing population growth rates, and *Daphnia magna* showed increasing mortality, in response to increasing levels of nZVI, and to a lesser degree with increasing Fe^2+^ and Fe^3+^. All forms of nZVI aggregated in soil and water, especially in the presence of a high concentration of calcium ions in groundwater, thus reducing their transports through the environment. However, uncoated nZVI aggregated extremely rapidly, thus vastly reducing the probability of environmental transport and potential for toxicity. This information can be used to design a risk management strategy to arrest the transport of injected nZVI beyond the intended remediation area, by injecting inert calcium salts as a barrier to transport.

## Introduction

Zero valent iron (ZVI) is an excellent electron donor that is used to transform via reduction or indirect oxidation many common contaminants in soil and groundwater [1]. The development of stable nano-scale ZVI (nZVI) products has generated significant interest in environmental remediation applications, with at least 80 pilot and field scale studies completed or underway [2]. Stable refers to the incorporation of a coating to the nZVI that reduces the rate of aggregation [3]–[9] and may also slow down the rate of release of Fe^2+^ from the core ZVI [4], [9]–[15]. Maintaining a stable small particle diameter is important to achieve sufficient mobility to reach the target contaminants. Reducing the rate of oxidation maximizes the electrons that are donated for the intended reactions. A number of commercial ZVI products are now available that contain stabilized nanoparticles.

While nZVI holds considerable promise for many remediation applications, the environmental risks are still poorly understood. In particular, the bioavailability and ecotoxicity of nZVI in different environmental media has not been studied in detail, and what we understand about ZVI toxicity is based mainly on studies of non-nano ZVI, or Fe^0^. There is an implicit assumption that nZVI is relatively non-toxic because Fe^0^ simply oxidizes to Fe^2+^ and then to Fe^3+^, both of which are common chemical species in the environment that most organisms are well adapted to deal with. However, ZVI applications can increase the concentration of Fe^2+^ and/or Fe^3+^ substantially at a local level in the short term. ZVI oxidation can also lead to the production of reactive oxygen species (ROS), such as hydroxyl radicals (OH·) from superoxide (O_2_·^−^) and hydrogen peroxide (H_2_O_2_) in living cells [16]. Fe ions enter the cytoplasm of cells and induce oxidative stress, which, among other impacts, can damage cell membranes leading to leakage of intracellular contents and cell death [17].

To date, only a few studies have evaluated the toxicity of nZVI, and most have focused on microbes (See Table 1 for summary). Uncoated nZVI, 35 nm (range 10–80 nm) in diameter, were toxic to *Escherichia coli* (ATCC strain 8739), displaying greater toxicity in hypoxic than aerobic conditions in soils and water [17]. Lee *et al.* also determined that ROS generation was responsible for *E. coli* death when exposed to Fe^2+^ at 5.6 mg L^−1^ Fe, but it required ≥56 mg L^−1^ Fe^3+^ to kill *E. coli*. Another study found mechanisms other than ROS by which nZVI can kill soil-based *E. coli*, including mitochondrial membrane damage, but also revealed that toxicity declines with the length of exposure because of a strong tendency for nZVI to form large aggregates (320±30 nm), regardless of soil pH [18]. Uncoated nZVI with 7–28% Fe^0^ content is toxic to *E. coli* at a concentration of about 5 mg L^−1^, but toxicity was not observed below 100 mg L^−1^ for humic acid coated-nZVI; below 140 mg L^−1^ for polyaspartate-coated nZVI; and below 516 mg L^−1^ for poly(styrene\sulfonate)-coated nZVI, thereby indicating that electrostatic repulsion provided by negatively charged coatings inhibits toxicity [7]. Other forms of toxicity, or a lack thereof, have been identified for fungi [18], viri [19], human cells [20] and rodent cells [12]. In aquatic ecosystems, polyaspartate-coated nZVI was found toxic to the amphidromous (seawater-freshwater inhabiting) medaka fish at concentrations beginning at ∼5 mg L^−1^ [16].

**Table 1:** Summary of previous studies of ZVI toxicology.

Organism	nZVI Type	Effects	Notes	Source
E. coli (ATCC strain 8739)	Uncoated 35 nm nZVI	90 mg L^−1^ (inactivation)	under aerated conditions	17
E. coli (ATCC strain 8739)	Uncoated 35 nm nZVI	9 mg L^−1^ (inactivation)	under deaerated conditions	17
E. coli (ATCC strain 8739)	Fe^2+^	5.6 mg L^−1^ Fe (inactivation)		17
E. coli (ATCC strain 8739)	Fe^3+^	56 mg L^−1^ (inactivation)		17
E. coli (Qc1301)	Uncoated 50 nm nZVI	7 mg L^−1^	Studies conducted at pH 5–5.5, toxicity observed after 1 hour contact	18
E. coli (ATCC strain 33876)	Uncoated nZVI	5 mg L^−1^ (MIC)		7
E. coli (ATCC strain 33876)	poly(styrene sulfonate) coated nZVI	516 mg L^−1^		7
E. coli (ATCC strain 33876)	polyaspartate coated nZVI	140 mg L^−1^		7
E. coli (ATCC strain 33876)	humic acid coated nZVI	100 mg L^−1^		7
B. subtilis	nZVI	1 g L^−1^	gram positive under aerobic conditions	19
P. fluorescens	nZVI	0.1 g L^−1^	gram negative under aerobic conditions	19
A versicolor	nZVI	No Effect	Survival ranged from 90–100% at alltested concentrations	19
MS-2 colphase virus	Uncoated 35 nm nZVI	0.1 mM		20
MS-2 colphase virus	Fe^2+^	0.1 mM	inactivation within 5 minutes	20
O. latipes embryos	Polyaspartate coated 30 nm nZVI	5 mg L^−1^ (enzymaticactivity changes)	pH 7–7.6. toxic effects observed withinthe first half day	16
O. latipes adults	Polyaspartate coated 30 nm nZVI	5 mg L^−1^	pH 7–7.6. gill samples showed increasing deposition of black particles, swelling ofthe epithelium cells and missing scales	16
M. galloprovincialis	50 nm Fe_2_O_3_	>10 mg L^−1^	no significant effect observed on development at varied pH levels	22
M. galloprovincialis	FeCl_3_	>0.8 mg L^−1^	no significant effect observed	22

Here we extend our understanding of the potential ecological risks of nZVI and its chemical byproducts to aquatic biota, specifically those inhabiting freshwater streams and coastal marine/estuarine waters, ecosystems that are connected to nZVI remediation sites via the seepage of groundwater [19]. We focus on the potential toxicity to primary producers, specifically phytoplankton (40–80 µm in size), and a primary consumer *Daphnia magna*, a freshwater zooplankton herbivore (1–2 mm). We chose these species because planktonic species are ecologically important as basal species in aquatic food webs, and are at substantial ecological risk from nanomaterial (NM) exposure due to potential exposure associated with terrestrial runoff or groundwater seepage into freshwater stream and ponds, as well as coastal bays, lagoons, and estuaries [21]. In addition, metal ions dissolved from some NMs may be readily bioavailable and harm phytoplankton cells, leading to declines in population growth rates and abundance [21]. Dissolution rates, and therefore the effective toxicity of NMs, often decreases with increasing ionic strength of the surrounding aquatic media (freshwater or seawater) because it leads to nanomaterial aggregation. The presence of ions also increases the rate of NM sedimentation, decreasing exposure to pelagic organism while increasing the probability of exposure to benthic organisms [22].

In light of these complex biological and chemical features, predicting under what conditions nZVI poses risks to planktonic organisms is challenging. To meet this important environmental challenge, we tested the following hypotheses: (1) the aggregation rate and aggregate size of nZVI varies with the type of coating, in the rank order of uncoated nZVI >polymer coated-nZVI > iron oxide coated-nZVI; (2) the aggregation rate and aggregate size for all forms of nZVI is greater in seawater than in freshwater; (3) following the aggregation behavior, the toxicity of nZVI is greater in freshwater than in seawater; and (4) based on oxidative capacity, toxicity of polymer coated-nZVI > iron oxide coated- nZVI > Fe^+2^> Fe^+3^. Uncoated nZVI was not used for the toxicity studies due to excessive aggregation which would have thoroughly confounded our results.

## Results

### Particle Size Analysis

**Figure 1:**
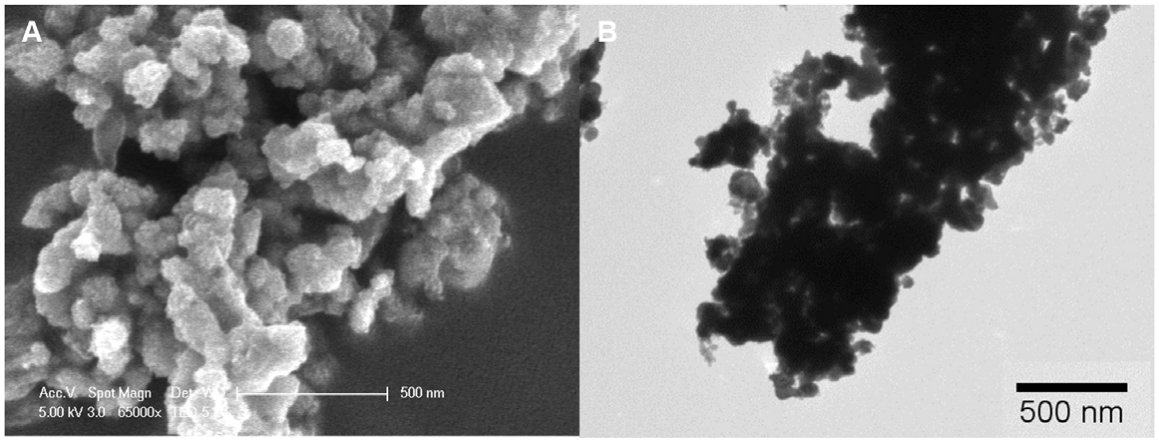
Nanofer 25S particles imaged with SEM and Nanofer STAR particles imaged with TEM.

SEM imaging of Nanofer 25S revealed aggregates of primary nano zero valent iron (nZVI) of approximately 80–120 nm diameter (Figure 1). Since the material was received as a slurry, the sample was dried before SEM imaging; thus the aggregation in the SEM images may not accurately reflect the size of the original material. Nanofer STAR was composed of aggregates of nZVI of ∼100–200 nm in diameter (Figure 1). The Nanofer 25, an uncoated material, appeared more agglomerated (see Figure S1 in Supporting Information), making it more difficult to determine the primary particle size.

### Particle Aggregation

**Figure 2:**
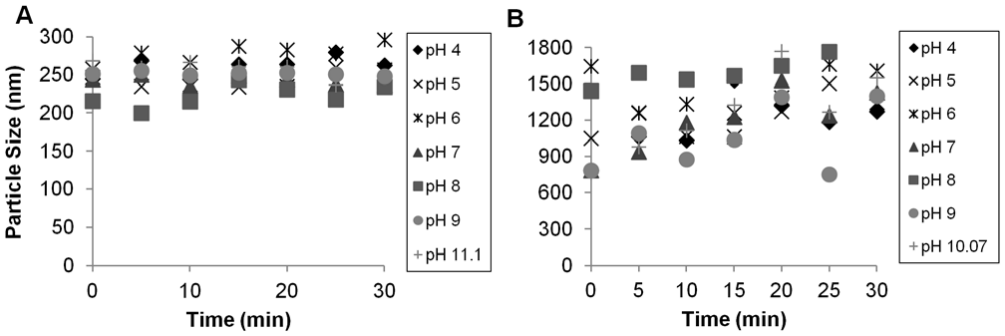
Nanofer 25S and Nanofer STAR particle size as a function of pH at 100 mg L^−1^, over time.

**Figure 3:**
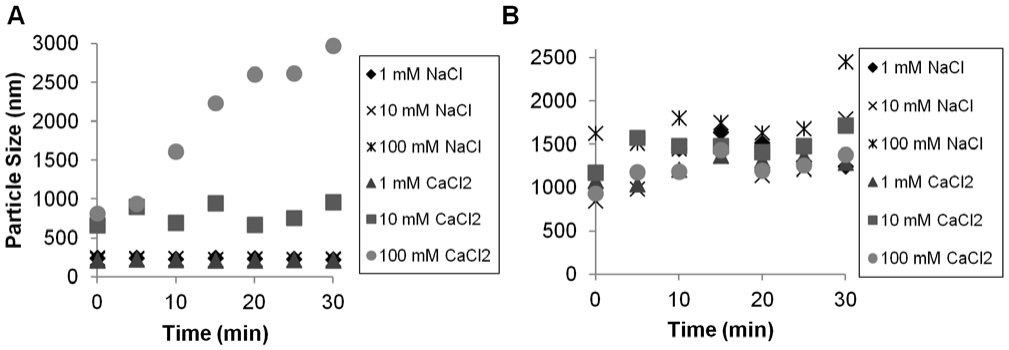
Nanofer 25S and Nanofer STAR particle size as a function of ionic strength at 100 mg L^−1^ and pH 7, over time.

We evaluated particle aggregation in synthetic water and natural (freshwater, groundwater, seawater) conditions. Nanofer 25S particles were stable at a hydrodynamic diameter (peak intensity) of around 250±50 nm across a range in pH from 4–11 (Figure 2A). Nanofer STAR particles were also relatively stable between 700 and 1800 nm across the same pH range (Figure 2B). The Nanofer 25 particles were rather large (several µm) at any pH from 4 to 10.5 (Figure S2). These experiments were conducted at low ionic strength (<1 mM). At higher ionic strength (IS), the Nanofer 25 particles started out very large (>3 µm), but remained fairly stable in size (Figure S3). There was not much difference between a monovalent ionic solution (NaCl) and a divalent ionic solution (CaCl_2_). Nanofer 25S aggregates, in contrast, remained stable even at high IS when NaCl was used, but began aggregating rapidly when CaCl_2_ was used to increase the IS to 10 or 100 mM (Figure 3A). At higher IS, the size of the Nanofer STAR particles remained relatively constant over the 30-minute experiments, though they were somewhat larger at higher ionic strengths (Figure 3B), regardless of the nature of the cations present. Thus, rapid aggregation in hard groundwater would be expected for all particles, particularly for Nanofer 25S, since its initial particle size is much smaller than for the other materials.

**Figure 4:**
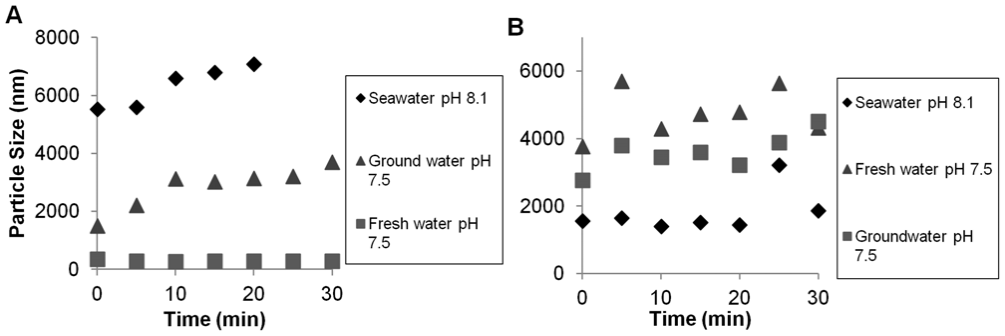
Nanofer 25S and Nanofer STAR particle size in different waters at 100 mg L^−1^, over time.

In the majority of natural water samples studied here aggregation of the particles was enhanced compared to the synthetic waters. Nanofer 25 particles aggregated to >3 µm very rapidly in the three natural water samples, and generally exhibited further aggregation over time (Figure S4). The Nanofer 25S particles were stable in freshwater (pH 7.5) at an aggregate size of around 280±50 nm, but aggregated in groundwater and seawater (Figure 4A). The Nanofer STAR particles were somewhat stable in freshwater and seawater, but formed large aggregates in other water samples (Figure 4B).

Generally, the zeta potential of the Nanofer 25 particles varied and was closer to neutral than the zeta potential of the Nanofer 25S particles, which was near −40 mV (Table S1). The zeta potential of the Nanofer STAR was around neutral, similar to the Nanofer 25, but with greater variation (Table S1). Neutral particles tend to aggregate faster unless a stabilizing coating is added to the nZVI. Aggregation of the particles was high when the charge was small, below around ±15 mV. Thus, the zeta potential of particles in a given media can be used to predict whether the particles will be stable or not.

### Toxicity to Phytoplankton

**Figure 5:**
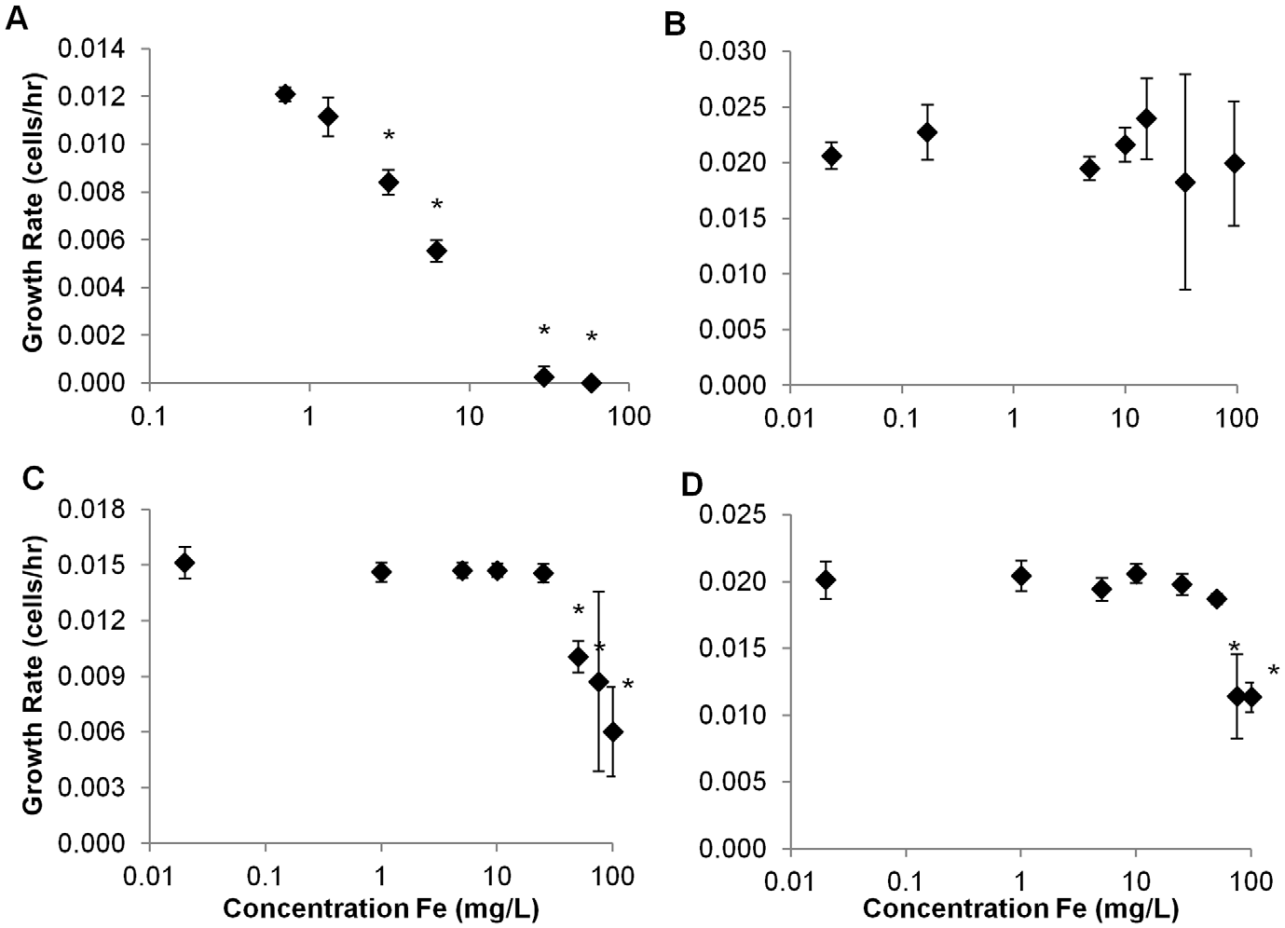
Growth Rate for *I. galbana* exposed to (a) Nanofer 25S, (b) Nanofer STAR, (c) Fe^2+^, and (d) Fe^3+^.

**Figure 6:**
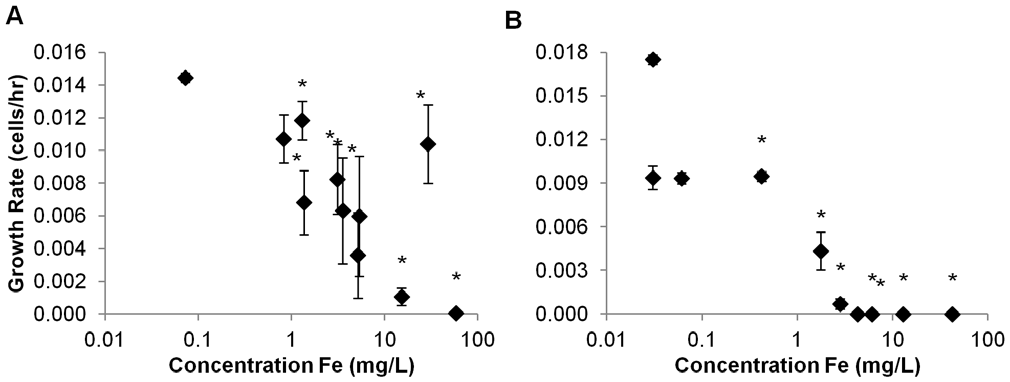
Growth Rate for (a) *D. tertiolecta* and (b) *T. pseudonana* exposed to Nanofer 25S.

**Figure 7:**
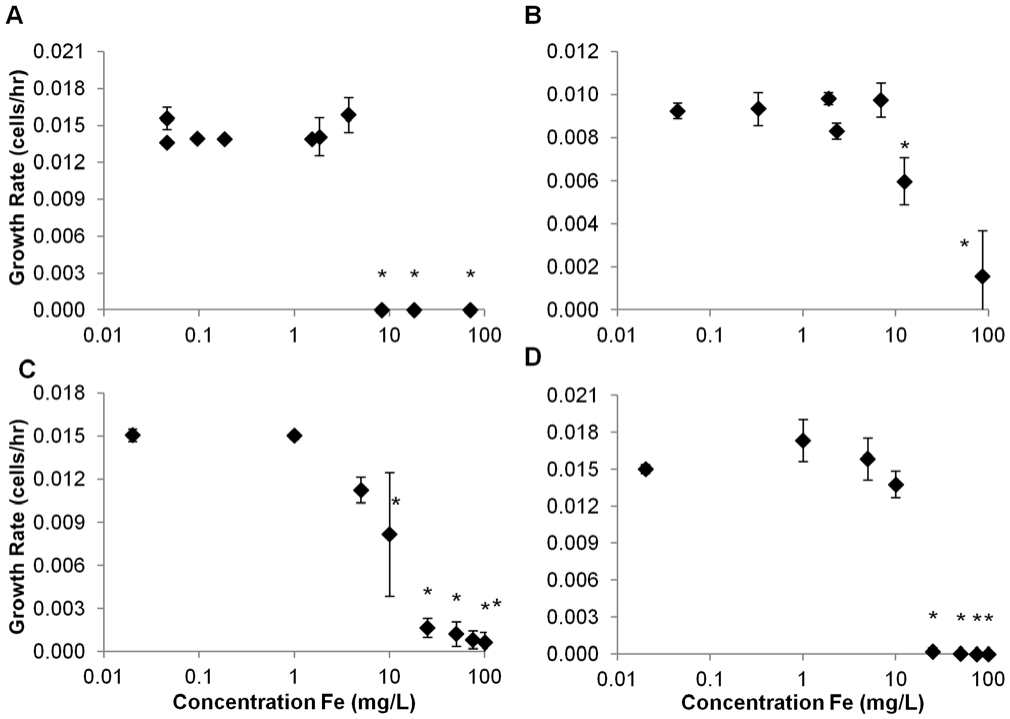
Growth Rate for *P. subcapitata* exposed to (a) Nanofer 25S, (b) Nanofer STAR, (c) Fe^2+^, and (d) Fe^3+^.

Population growth of the marine phytoplankton species *Isochrysis galbana* was significantly depressed at concentrations of Nanofer 25S ≥3 mg L^−1^ (Figure 5A) compared with controls. Growth was reduced to near zero above 6 mg L^−1^ (Figure 5A). In contrast, growth of *I. galbana* was not significantly affected by Nanofer STAR at any concentration (Figure 5B). Ionic iron species did not reduce growth of *I. galbana* at concentrations below 50 mg L^−1^ for Fe^2+^ and below 75 mg L^−1^ for Fe^3+^ (Figures 5C, D). Since it exhibited the highest toxic potential of the two particles, we tested the effects of Nanofer 25S on two additional species of marine phytoplankton, *Dunaliella tertiolecta* and *Thalassiosira pseudonana*. Population growth of both species was depressed at low concentrations of this nanomaterial: 1.3 mg L^−1^ for *D. tertiolecta* and 0.4 mg L^−1^ for *T. pseudonana* (Figure 6). Population growth of the freshwater phytoplankton species *Pseudokirchneriella subcapitata* was not significantly affected by Nanofer 25S at concentrations <8 mg L^−1^ (Figure 7A). However, unlike the case for *I. galbana*, Nanofer STAR significantly impacted *P. subcapitata* at concentrations ≥12 mg L^−1^ (Figure 7B). *P. subcapitata* was also more sensitive to Fe^2+^ and Fe^3+^, which significantly reduced its growth rate at concentrations of 10 mg L^−1^ for Fe^2+^ and 25 mg L^−1^ for Fe^3+^ (Figures 7C, D).

### Toxicity to Zooplankton

**Figure 8:**
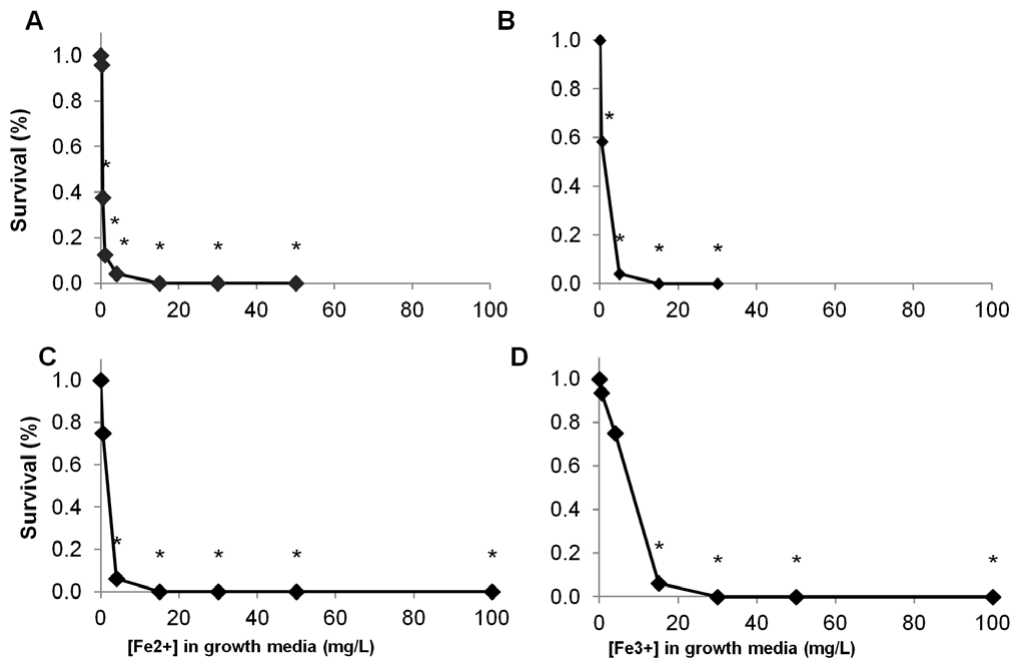
*D. magna* survival after 4 days in the presence of (a) Nanofer 25S, (b) Nanofer STAR, (c) Fe^2+^, and (d) Fe^3+^.

**Figure 9:**
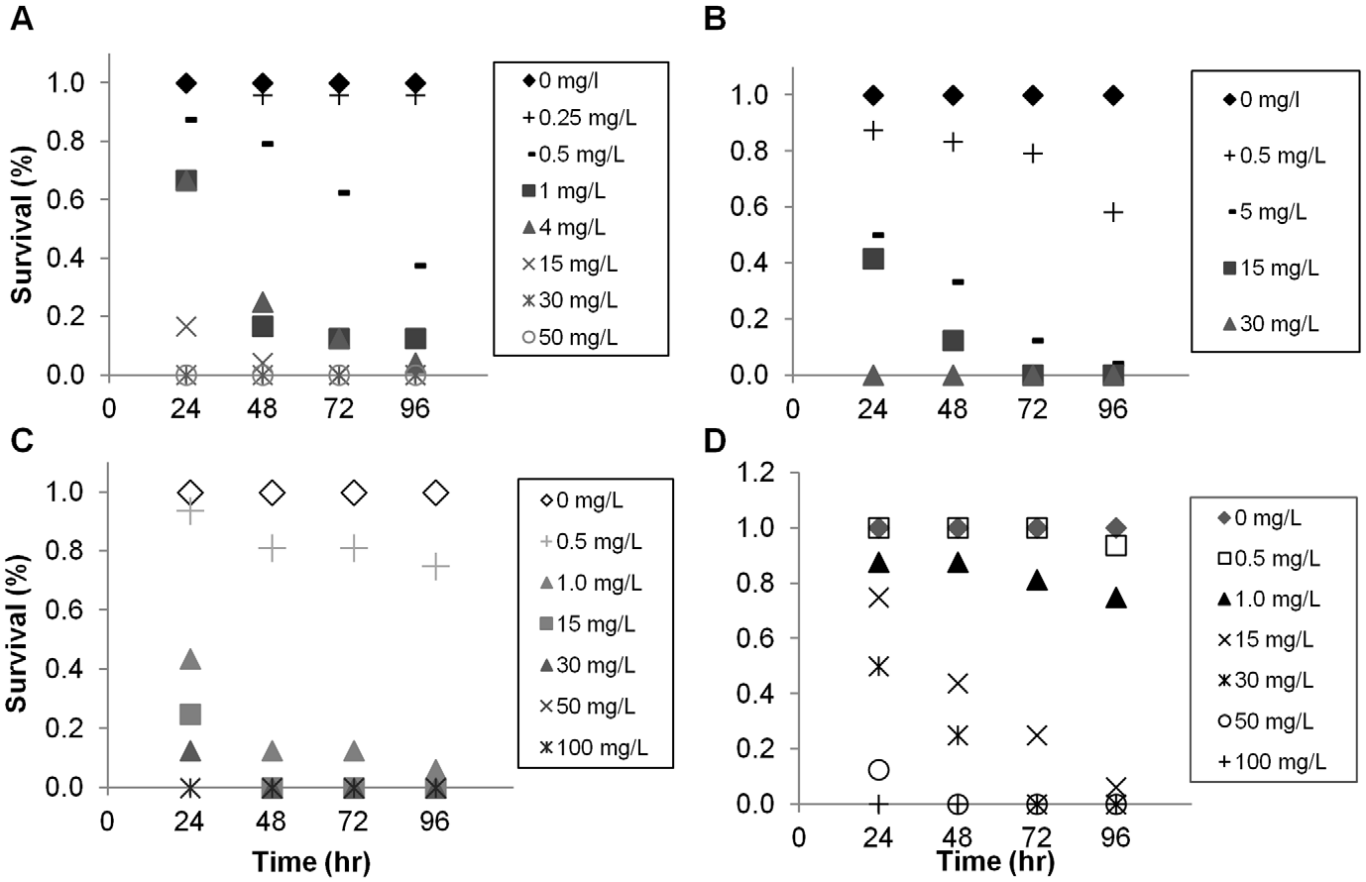
*D. magna* survival over time in the presence of Nanofer 25S and Nanofer STAR at different concentrations, measured as total Fe.

Exposure tests (96-hour) showed that *Daphnia magna* survival was dramatically impacted by both Nanofer 25S and Nanofer STAR at total Fe concentrations ≥0.5 mg L^−1^ (Figures 8A, B). To determine whether the observed toxicity for the nZVI was attributable only to nanoparticle-associated Fe^(0)^, we evaluated the toxicity to *Daphnia magna* of Fe^2+^ and Fe^3+^ amended growth media (Figure 8C, D). Higher concentrations of Fe^2+^ and Fe^3+^ were reached before significant mortality effects: 4 mg L^−1^ for Fe^2+^ and 15 mg L^−1^ for Fe^3+^, although there were indications of decreased survival at the lowest concentrations also (Figures 8C, D). Daily survival data as the experiments progressed indictated that at concentrations above ∼1 mg L^−1^ Nanofer particles caused significant die-offs within the first 24–48 hours (Figure 9A,B), and a similar response was observed for Fe^2+^ (Figure 9C). *D. magna* responded more slowly to Fe^3+^ at all but the highest concentrations >15 mg L^−1^ (Figure 9D).

## Discussion

Our results show that commercial formulations of nZVI can be toxic to aquatic organisms that may be exposed to the material downstream of remediation sites, either in freshwater streams, ponds, or in the coastal marine environment (Table 2). These values represent the no observed effect concentration (NOEC), and an assessment factor would have to be applied to estimate the Predicted No Effect Concentration (PNEC) based on OECD’s guidelines. For reference, we summarize the previous results with nZVI and related materials. We found that the toxicity is strongly dependent on the form of nZVI, which is an important consideration when using these nanomaterials in remediation applications. Coatings can profoundly affect the toxicity of nZVI, and in general we found support for our first two hypotheses that (1) the aggregation rate of uncoated nZVI >polymer coated-nZVI > iron oxide coated-nZVI, and (2) that the aggregation rate for all forms of nZVI is greater in seawater than in freshwater. Uncoated particles, in this case Nanofer 25, aggregated so rapidly as to make them unsuitable for remediation applications. Nanofer 25S, which was coated with polyethylene glycol sorbitan monostearate, showed minimal aggregation in pH 7.5 freshwater, although aggregation of this particle was still generally high in hard groundwater (Figure 4). Nanofer STAR was capped with a 2 nm Fe-O shell, and although it was stable, the initial aggregate size was large in all tested media (Figure 4). The latter two particles aggregated in seawater, as expected, but aggregation of the surfactant-coated Nanofer 25S was faster than for Nanofer STAR (Figure 4).

**Table 2:** Summary of results of aggregation and toxicity studies.

Material	Nanofer 25	Nanofer 25S	Nanofer STAR	Fe^2+^	Fe^3+^
Coating	Uncoated	Polyethylene Glycol Sorbitan Monostearate	2 nm Iron Oxide Shell	None	None
Initial Aggregate Size	>500 nm	80–120 nm	100–200 nm		
Aggregation at pH 7, 30 min	4359	237.2	1431		
Statistically Significant Toxic Effect*					
*I. galbana*	ND	3.1 mg L^−1^	>100 mg L^−1^	50 mg L^−1^	75 mg L^−1^
*D. tertiolecta*	ND	1.3 mg L^−1^	ND	ND	ND
*T. pseudonana*	ND	0.42 mg L^−1^	ND	ND	ND
*P. subcapitata*	ND	8.24 mg L^−1^	12.4 mg L^−1^	5 mg L^−1^	25 mg L^−1^
*D. magna*	ND	0.5 mg L^−1^	0.5 mg L^−1^	1 mg L^−1^	15 mg L^−1^

* Results are for observed statistically significant toxic effect.

As predicted, the smaller aggregate size of Nanofer 25S increased its toxicity over Nanofer STAR [23]; the passivated iron oxide surface of the Nanofer STAR also reduces its reactivity and toxicity. The NOEC of Nanofer 25S on population growth of the freshwater phytoplankton *P. subcapitata* was 8.2 mg L^−1^ versus 12.4 mg L^−1^ for Nanofer STAR (Figure 6). Both particles were highly toxic to *D. magna* at low concentrations, although Nanofer 25S induced mortality more rapidly (Figures 8 and 9). We expected that the high level of aggregation of the nZVI in salt water would result in lower toxicity of the particles, but that was not always the case. Indeed, Nanofer 25S significantly depressed growth of the marine microalgae *I. galbana* at concentrations as low as 3 mg L^−1^; Nanofer STAR, however, showed no effect on *I. galbana* even at very high concentrations close to 100 mg L^−1^, suggesting that aggregation in seawater did affect its toxicity (Figure 5). Exposure tests with two additional species of marine phytoplankton also showed toxicity of Nanofer 25S at relatively low concentrations. Since the pH remained fairly constant throughout the exposures (Table S2, freshwater range from 7.5–8.1 and seawater range from 8.1–8.3), it is unlikely to be a factor in the toxicity. However, light transmission did decrease by around 10% for the concentration of iron around 10 mg L^−1^ and more than 95% when the concentration of iron is greater than 100 mg L^−1^, which can be an important factor affecting growth of phytoplankton at these higher iron concentrations (Figure S5). However, when the concentration of iron is less than 5 mg L^−1^, this mechanism is likely to be minor or negligible in overall toxicity.

We predicted that the hierarchy of oxidative capacity among the forms of Fe tested, organic coated-nZVI (Nanofer 25S) > iron oxide coated nZVI (Nanofer STAR) > Fe^+2^> Fe^+3^, would be mirrored in the toxicity results. In general this was the case. Nanofer 25S consistently exhibited toxicity at lower concentrations than either Nanofer STAR or ionic Fe in both freshwater and marine organisms. Fe^+2^ consistently showed toxicity at lower concentrations than Fe^+3^. Nanofer STAR, however, did not always show higher toxicity than Fe^+2^; indeed, this particle showed no evident toxic effects on growth rate of the marine phytoplankton species *I. galbana* and its toxicity to the freshwater microalgae *P. subcapitata* was lower than that for Fe^+2^ (Figure 6). Nevertheless, Nanofer STAR was highly toxic to the freshwater suspension-feeder *D. magna*, causing mortality at a much lower dose than Fe^+2^ or Fe^+3^ (Figure 8). Two potential explanations for decreased toxicity of metal oxide nanoparticles with increasing charge have been put forth: 1) the corresponding increase in energy needed for release of dissolved ions, and 2) the decrease in ionization potential with increasing charge [24]. The first scenario is unlikely in this case, since the nanoparticles in general exhibited toxicity at lower concentrations than the salts despite their particulate nature. Ionization potential, and the increased power of Fe to catalyze production of hydroxyl radicals with lower charge, is more likely the cause of the relationship seen here. Intracellular iron, indeed, has been shown to be a potent cause of hydrogen peroxide-induced DNA damage [25].

Our focus in this study were freshwater and marine organisms that may be exposed to groundwater with residual yet elevated concentrations of nZVI, Fe^2+^ and Fe^3+^. Other work on aquatic organisms is thus far fairly limited. nZVI had a significant impact on medaka (*Oryzias latipes*) fish and their embryos [16]. Commercial nZVI (primary size 30 nm) coated with 4 wt% of sodium polyaspartate were used at concentrations ranging from of 0.5 and 50 mg L^−1^ of nZVI, at pH 7 to 7.6, with a hardness of 200 mg L^−1^ as CaCO_3_. The embryos exhibited changes in enzymatic activity in response to the ROS at 5 and 50 mg L^−1^; even after only 0.5 day exposure, with increasing changes in enzymatic activity as the exposure time increased to 8 days [16]. In the adults, gill samples showed increasing deposition of black particles, swelling of the epithelium cells and missing scales at concentrations of 5 and 50 mg L^−1^, after 14 days of exposure. Swelling and black particle accumulation was also observed in the intestines at these higher concentrations. No effect was observed in liver or brain cells. Under natural 0.2 µm-filtered seawater conditions, no significant effect was observed for 50 nm Fe_2_O_3_ nanoparticles or an FeCl_3_ solution on the development of a mussel, *Mytilus galloprovincialis*, at varied pH levels [26] and concentrations up to 10.0 mg L^−1^ for the ferric oxide nanoparticles or 0.80 mg L^−1^ of FeCl_3_.

Although there was clearly a toxic effect from dissolved Fe^2+^ and Fe^3+^, the nZVI exhibited additional toxicity due perhaps to the nanoparticles, their aggregates, or the H_2_ released during the transformation of the nZVI. In most cases, the response at 1 mg L^−1^for ferrous and ferric iron was not statistically different from the control, and even the effect at 5 mg L^−1^ and in some cases even 10 mg L^−1^ were not as deleterious as observed from Nanofer 25S. It is likely that the nZVI attaches to the cell surfaces and transfers electrons to different biochemicals at the surface, leading to undesired reactions. The concentration-response curves based on the growth rate of the phytoplankton population indicated that the marine phytoplankton species *I. galbana* was more tolerant of either Fe^2+^ or Fe^3+^ than the freshwater algae *P. subcapitata*. The growth rate of *I. galbana* was statistically the same as the control (lowest Fe, present in seawater) up to around 15 mg L^−1^for either Fe^2+^ or Fe^3+^ (Figure 5). However, *I. galbana* tolerated Fe^3+^ better than Fe^2+^. In the case of *P. subcapitata*, effects were noticeable at >1 mg L^−1^for both Fe^2+^or Fe^3+^, and there was almost no difference between the iron species (Figure 6).

### Conclusions

Given that pilot and full remediation tests have used concentrations of approximately 4.5 to 300 g L^−1^ of nZVI slurry [27], the concentrations that one may expect in the aquatic environment influenced by the discharge from a remediation site could range from µg L^−1^ to mg L^−1^. Nanofer 25S exhibited toxicity at 0.5–1.0 mg L^−1^ in freshwater media to the freshwater phytoplankton *P. subcapitata* and the water flea *D. magna*, and was also toxic for three species of marine phytoplankton at 0.3–3.0 mg L^−1^, similar to the case for freshwater. The toxicity likely stems in part from the oxidation products released from the ZVI particles, namely Fe^2+^ and Fe^3+^ ions. Additional studies may show that at the surface of the interaction between the ZVI and the organisms, oxidation reactions from the oxidation of Fe^(0)^ to Fe^2+^ also result in localized damage which can ultimately affect growth and even survival. In many cases nZVI will be injected into the subsurface at a significant distance from freshwater or coastal receptors, resulting in considerable dilution of the concentrations of Fe^2+^ and Fe^3+^ ions, or precipitation of iron compounds. However, it would be important to monitor the concentration of these ions downgradient from an nZVI injection site, to determine whether there is sufficient dilution or precipitation. Uncoated nZVI aggregate too rapidly to transport significantly, but even nZVI with either an organic surfactant coating or an iron oxide protective layer tend to aggregate with time, particularly in the presence of a high concentration of calcium ions in hard groundwater. This information can be used to design a risk management strategy to arrest the transport of injected nZVI beyond the intended remediation area, by injecting Ca salts as a barrier to transport.

## Methods

### Materials

Three commercial nZVI were evaluated, Nanofer 25, Nanofer 25S, and Nanofer STAR (all from NANO IRON s.r.o., Rajhrad, Czech Republic). The materials were received by air shipment, with Nanofer 25 and Nanofer 25S as aqueous suspensions, and Nanofer STAR as a powder. According to the manufacturer, the iron content of all three Nanofers is 70–90% nZVI and 10–30% iron oxides when produced. Nanofer 25S is coated with polyethylene glycol sorbitan monostearate, a surfactant. Nanofer STAR particles are coated with 2 nm iron oxide shell to reduce their oxidation, allowing *in-situ* preparation of the suspensions.

### Particle Size and Aggregation Studies

The size of the nZVI was determined using Scanning Electron Microscopy (SEM) and Dynamic Light Scattering (DLS). Particles were imaged using aZL40 Sirion FEG Digital Scanning Microscope w/EDS (FEI, USA). Aggregation studies using DLS (Zetasizer, Malvern Instruments, Ltd., UK) were conducted over 120 min periods in different waters, including a surface water, groundwater and seawater. Groundwater was considered to understand the potential mobility of the nZVI after injection. Freshwater and seawater were considered in the toxicity studies. Since the initial pH of the freshwater and groundwater were low, aggregation was studied at an adjusted pH of 7.5 using 0.1 M NaOH. A detailed characterization of these waters is provided in the File S1. The charge on the ZVI particles was also measured using the Zetasizer.

### Toxicity Studies

Three species of marine phytoplankton were used: *Thalassiosira pseudonana* (centric diatom, Bacillariophyceae: Centrales); *Dunaliella tertiolecta* (Chlorophyceae: Chlamydomonadales), and *Isochrysis galbana* (Prymnesiophyceae: Isochrysidales). Axenic cultures were obtained from the Provasoli-Guillard National Center for Culture of Marine Phytoplankton (Bigelow Laboratory for Ocean Sciences, West Boothbay, Harbor, Maine, USA), and were maintained in standard media (f/2, 23, 24) made with 0.22 µm filtered natural seawater, which was autoclaved prior to inoculation. The background total Fe in the seawater media averaged 0.04 mg L^−1^±0.04 mg L^−1^. To provide inoculant for exposure experiments, the phytoplankton were incubated under cool white fluorescent lights (14∶10 light:dark) at 20°C with aeration for 5–7 days until growth reached log phase. Cell densities were measured by hemacytometer (Reichert, Buffalo, NY). Experiments were conducted at 20°C, 34 parts per thousand salinity (‰), under the same fluorescent lights. All equipment was acid-washed, rinsed with nanopure water, and autoclaved before use. For media, f/2 was used, [23], [24] with only major nutrients added and no trace metals, to avoid adding EDTA that would complex free metal ions. Cells to inoculate the experiments were first filtered (0.22 µm) and rinsed three times with filtered autoclaved seawater to remove EDTA, and resuspended in EDTA-free growth media. Experiments were run in 500 ml Erlenmeyer flasks, media volume 200 ml, and were mixed at ∼150 rpm on a rotary shaker (New Brunswick Scientific Co., NJ, USA). The nZVI concentrations tested ranged from 0.2 to 100 mg L^−1^ total Fe, with five replicates per treatment. Five replicates per nZVI treatment were conducted. Flasks were inoculated with 1–2 · 10^5^ cells ml^−1^. Cell densities were monitored every 24 hours for 96 hours by fluorometer (Trilogy, Turner Designs, Sunnyvale, CA).

One species of freshwater phytoplankton, *Pseudokirchneriella subcapitata* (Chlorophycea: Sphaeropleales) was tested. Starter cultures were obtained from Carolina Biological Supply (Burlington, NC, USA), and were maintained in standard freshwater media [27] made with ultrapure filtered water (0.2 µg, Nanopure), which was autoclaved prior to inoculation. All other conditions were the same as for the marine phytoplankton. The background concentration of total Fe in the media was 0.01 mg L^−1^.

Nanofer 25 aggregated so rapidly (see Results) that it would not be useful in remediation, thus our toxicity studies focused on Nanofer 25S and Nanofer STAR. All four phytoplankton species (three marine and one freshwater) were exposed to Nanofer 25S, but only one marine (*I. galbana*) and the freshwater phytoplankton (*P. subcapitata*) were exposed to Nanofer STAR because we had a limited supply of the nanomaterials. We expected Nanofer to dissolve and produce dissolved iron, which is naturally present in freshwater and seawater. To test whether toxicity was due to the nanomaterial or the dissolved iron that accumulates in the media with the dissolution of the Nanofer, we compared toxicity of Nanofer 25S and Nanofer STAR and dissolved iron, which we mimicked using iron chloride salts (FeCl_2_ and FeCl_3_) at concentrations of Fe^2+^ and Fe^3+^. with one marine (*I. galbana*) and one freshwater (*P. subcapitata)* phytoplankton species.

Toxicity for phytoplankton was measured as a reduction in population growth rates, which were estimated for each replicate flask as the slope of log-transformed cell count data, obtained through least-squares regression [24]. One-way ANOVA was used to test for an overall effect of NP toxicity on growth rates. Homogeneity of variances was tested with Levene’s test, and when heterogeneous, data were transformed. When ANOVA revealed significant differences among treatments, post-hoc tests were conducted with Dunnett’s method [25], which tests for pair-wise differences between each treatment and the control.

The toxicity of the nZI to *Daphnia magna*, a freshwater zooplankton grazer, was tested by measuring the survival of young (neonate) individuals as per EPA Method 2021 [28]. Cultures of adult *D. magna* were obtained from Sachs Systems Aquaculture (St. Augustine, Fl). Pregnant females were separated until neonates were present, which were then collected and transferred to test Petri dishes. Duplicate studies were done for treatments 15 mg L^−1^ total Fe and below. Exposure was conducted by pipetting sufficient Nanofer 25S suspension to achieve the desired total Fe concentration from 0.2 to 100 mg L^−1^ total Fe. Survival of neonates was monitored daily for 96 hours.

To determine the concentration of total Fe in the test media, 4 ml of trace-metal-free nitric acid was added to a 1 ml sample of the media used for each phytoplankton trial. This sample was then digested in a HACH DRB200 digester (Hach, USA) at 80°C for 60 minutes and cooled for 30 minutes, diluted to 50 ml in a volumetric flask using nanopure water, and analyzed via ICP-AES (iCAP 6300, Thermo Scientific, Waltham, MA). NIST-traceable standard solutions for total Fe (Fluka Analytical, Switzerland) were used to generate calibration curves ranging from 0.01 to 100 mg L^−1^ for comparison.

## Supporting Information

Figure S1 **Nanofer 25 particles imaged with SEM. Scale is 500 nm.**
(TIF)

Figure S2 **Nanofer 25 particle size at a function of pH, over time.**
(TIF)

Figure S3 **Nanofer 25 particle size as a function of ionic strength, over time.**
(TIF)

Figure S4 **Nanofer 25 particle size in different waters, over time.**
(TIF)

Figure S5 **Transmission of light in freshwater and seawater for Nanofer 25S, STAR, and dissolved Fe^2+^ and Fe^3+^ at different nominal Fe concentrations.**
(PNG)

Table S1 **Initial particle charge (zeta potential) for different ZVI under different conditions.**
(DOCX)

Table S2 **Evolution of pH after addition of ZVI into freshwater and seawater.**
(DOCX)

File S1 (DOCX)
