# Supplementary figures and images for: Toxicity of Nano-Zero Valent Iron to Freshwater and Marine Organisms
Source: PLoS One. 2012 Aug 30;7(8):e43983. doi: 10.1371/journal.pone.0043983 (PMC3431385; doi:10.1371/journal.pone.0043983)

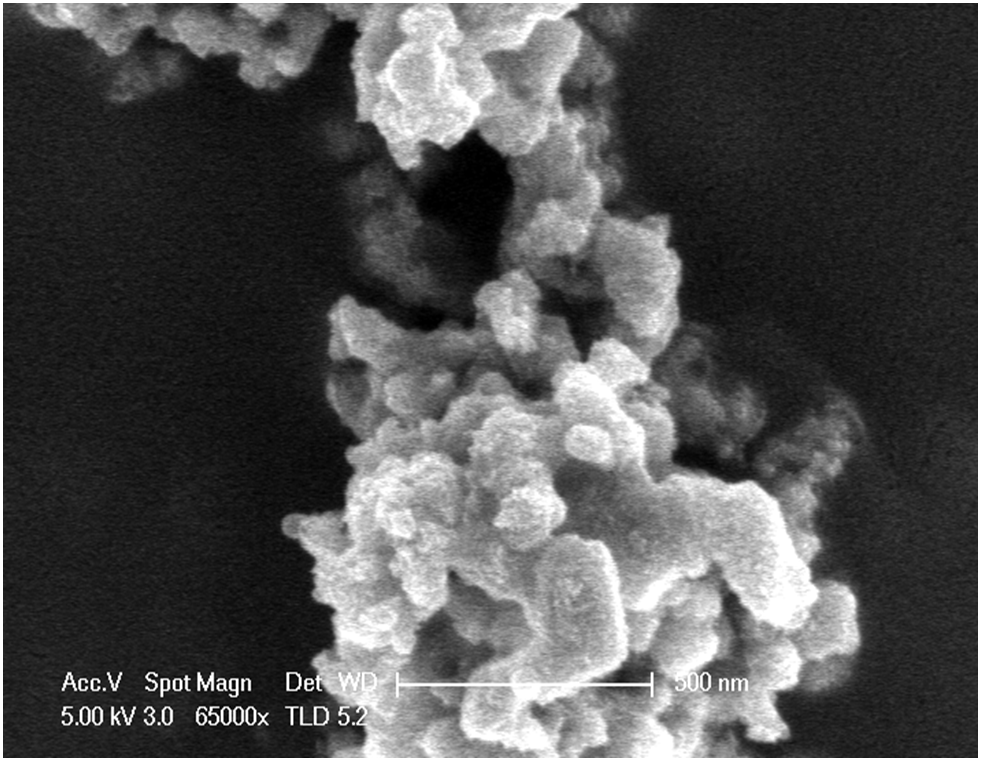

Supplement: Figure S1 — Nanofer 25 particles imaged with SEM. Scale is 500 nm. (TIF) [file pone.0043983.s001.tif]

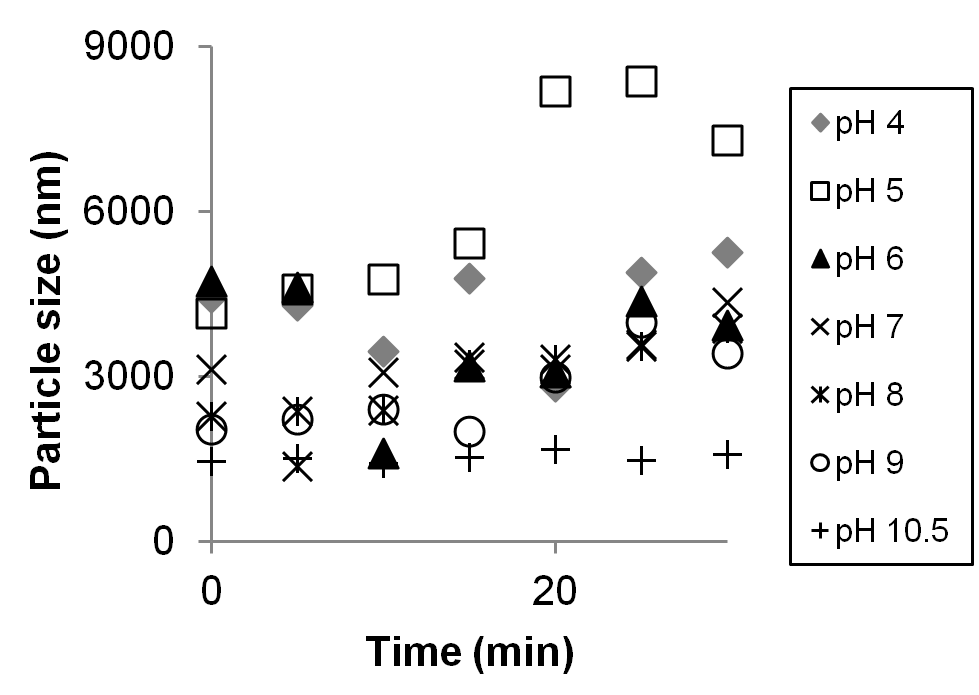

Supplement: Figure S2 — Nanofer 25 particle size at a function of pH, over time. (TIF) [file pone.0043983.s002.tif]

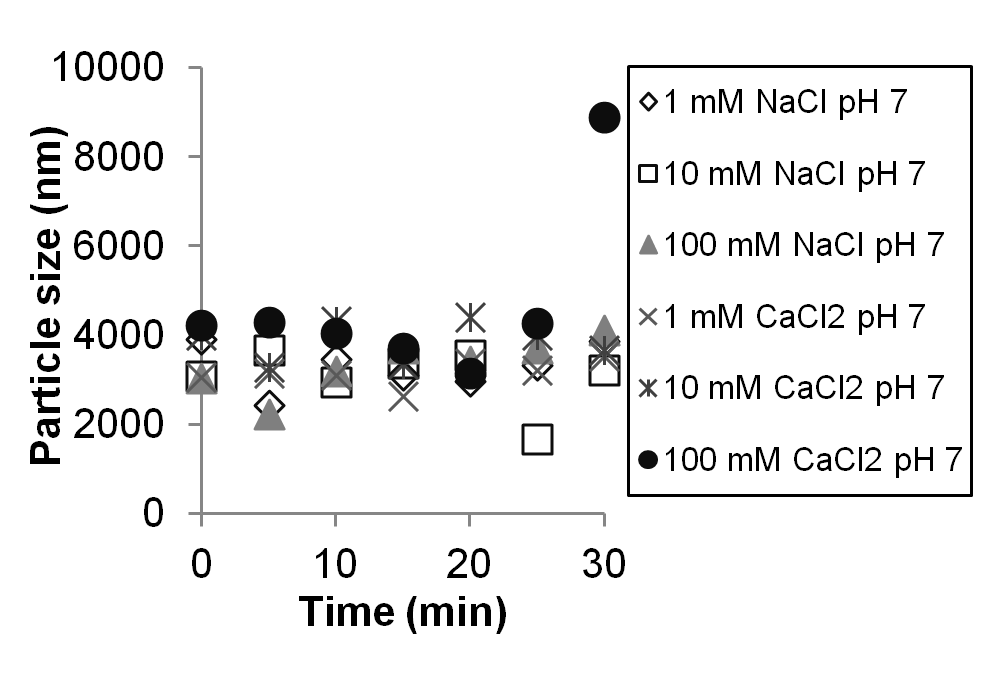

Supplement: Figure S3 — Nanofer 25 particle size as a function of ionic strength, over time. (TIF) [file pone.0043983.s003.tif]

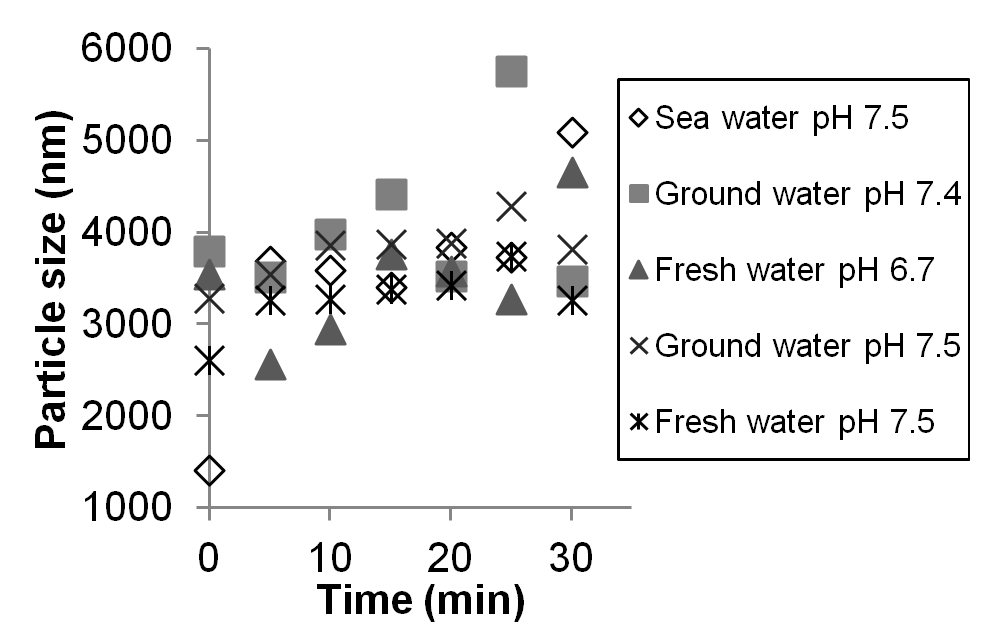

Supplement: Figure S4 — Nanofer 25 particle size in different waters, over time. (TIF) [file pone.0043983.s004.tif]

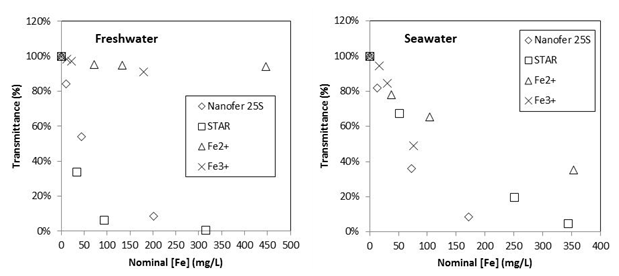

Supplement: Figure S5 — Transmission of light in freshwater and seawater for Nanofer 25S, STAR, and dissolved Fe2+ and Fe3+ at different nominal Fe concentrations. (PNG) [file pone.0043983.s005.png]
