# Supplementary material for: Toxicity of Nano-Zero Valent Iron to Freshwater and Marine Organisms
Source: PLoS One. 2012 Aug 30;7(8):e43983. doi: 10.1371/journal.pone.0043983 (PMC3431385; doi:10.1371/journal.pone.0043983)
Supplement: Table S1 — Initial particle charge (zeta potential) for different ZVI under different conditions. (DOCX) [file pone.0043983.s006.docx]

Table S1. Initial particle charge (zeta potential) for different ZVI under different conditions.

| **Material** | **Nanofer 25** | **Nanofer 25S** | **STAR** |
| --- | --- | --- | --- |
| pH 4 | 1 | -17.4 | 19.7 |
| pH 5 | -1.8 | -28.3 | 6.26 |
| pH 6 | -2.9 | -36.4 | -24.4 |
| pH 7 | -3.2 | -40.0 | -23.2 |
| pH 8 | -7.3 | -43.0 | -7.48 |
| pH 9 | -4.3 | -41.7 | -10.4 |
| 1 mM NaCl pH 7 | 8.5 | -41.7 | -17.1 |
| 10 mM NaCl pH 7 | 9.2 | -34.5 | 0.0945 |
| 100 mM NaCl pH 7 | 9 | -25.5 | -4.5 |
| 1 mM CaCl2 pH 7 | 16.5 | -33.7 | -5.18 |
| 10 mM CaCl2 pH 7 | 22 | -15.7 | -0.927 |
| 100 mM CaCl2 pH 7 | 14.7 | -2.5 | 2.77 |
| Sea water pH 7.5 | 3.5 | -13.5 | -0.96 |
| Ground water pH 7.5 | -1.6 | -1.9 | -3.32 |
| Fresh water pH 7.5 | 2 | 1.7 | -5.85 |
