# Supplementary material for: Toxicity of Nano-Zero Valent Iron to Freshwater and Marine Organisms
Source: PLoS One. 2012 Aug 30;7(8):e43983. doi: 10.1371/journal.pone.0043983 (PMC3431385; doi:10.1371/journal.pone.0043983)
Supplement: Table S2 — Evolution of pH after addition of ZVI into freshwater and seawater. (DOCX) [file pone.0043983.s007.docx]

Table S2. Evolution of pH after addition of ZVI into freshwater and seawater.

| Sample | Concentration Fe (mg/L) | 0 minutes | 30 minutes | 24 hours | 48 hours | 72 hours | 96 hours |
| --- | --- | --- | --- | --- | --- | --- | --- |
| Seawater | 0.13 | 8.23 | 8.23 | 8.22 | 8.23 | 8.22 | 8.23 |
| Freshwater | 0.04 | 8.18 | 8.35 | 8.30 | 8.25 | 7.75 | 7.86 |
| Sea Nanofer 25S | 1.34 | 8.25 | 8.23 | 8.24 | 8.20 | 8.23 | 8.11 |
| Sea Nanofer 25S | 2.88 | 8.26 | 8.24 | 8.28 | 8.22 | 8.21 | 8.18 |
| Sea Nanofer 25S | 19.30 | 8.31 | 8.28 | 8.31 | 8.22 | 8.22 | 8.21 |
| Sea Nanofer 25S | 42.41 | 8.31 | 8.26 | 8.30 | 8.22 | 8.22 | 8.20 |
| Sea Nanofer 25S | 144.20 | 8.30 | 8.35 | 8.34 | 8.34 | 8.30 | 8.21 |
| Fresh Nanofer 25S | 1.70 | 8.15 | 8.39 | 8.40 | 8.07 | 7.92 | 8.05 |
| Fresh Nanofer 25S | 3.96 | 8.13 | 8.15 | 8.30 | 8.08 | 7.82 | 8.04 |
| Fresh Nanofer 25S | 12.00 | 8.05 | 8.16 | 8.30 | 8.05 | 7.79 | 7.93 |
| Fresh Nanofer 25S | 27.85 | 7.78 | 7.95 | 8.25 | 8.10 | 7.85 | 7.95 |
| Fresh Nanofer 25S | 197.92 | 7.96 | 8.15 | 8.14 | 7.99 | 7.81 | 7.91 |
| Sea STAR | 1.00 | 8.24 | 8.23 | 8.19 | 8.18 | 8.12 | 8.05 |
| Sea STAR | 3.66 | 8.31 | 8.23 | 8.21 | 8.19 | 8.15 | 8.10 |
| Sea STAR | 15.22 | 8.33 | 8.27 | 8.23 | 8.20 | 8.16 | 8.14 |
| Sea STAR | 50.10 | 8.33 | 8.27 | 8.24 | 8.20 | 8.20 | 8.18 |
| Sea STAR | 183.23 | 8.54 | 8.68 | 8.34 | 8.33 | 8.33 | 8.30 |
| Fresh STAR | 0.97 | 8.07 | 8.04 | 7.94 | 7.97 | 7.95 | 7.90 |
| Fresh STAR | 2.81 | 7.96 | 7.87 | 7.78 | 7.77 | 7.76 | 7.75 |
| Fresh STAR | 21.71 | 7.77 | 7.78 | 7.72 | 7.69 | 7.68 | 7.70 |
| Fresh STAR | 48.70 | 7.51 | 7.60 | 7.62 | 7.56 | 7.60 | 7.66 |
| Fresh STAR | 140.32 | 7.37 | 7.59 | 7.60 | 7.45 | 7.52 | 7.59 |
