# Supplementary material for: Toxicity of Nano-Zero Valent Iron to Freshwater and Marine Organisms
Source: PLoS One. 2012 Aug 30;7(8):e43983. doi: 10.1371/journal.pone.0043983 (PMC3431385; doi:10.1371/journal.pone.0043983)
Supplement: File S1 — (DOCX) [file pone.0043983.s008.docx]

**Toxicity of Zero Valent Iron to Freshwater and Marine Organisms**

Arturo A. Keller, Kendra Garner, Robert Miller and Hunter S. Lenihan

University of California Center for Environmental Implications of Nanotechnology

Bren School of Environmental Science and Management, University of California Santa Barbara

Supporting Information

**Particle Size Analysis**

Scanning electron microscopy (SEM) image for Nanofer 25 is presented in Figure S1. The particles appeared more agglomerated, making it more difficult to determine the primary particle size.

**Aggregation Studies**

The Nanofer 25S quickly aggregated to micron scale particles (Figure S2) at all pH considered. Aggregation was more significant at low pH (4-6) but was still considerable even at higher pH (7-10.5).

At high ionic strength (IS) and pH 7, the Nanofer 25 particles were very large (> 3 µm) even at time zero, but remained fairly stable in size (Figure S3).

Under most natural conditions, the Nanofer 25 particles aggregated to > 3 µm very rapidly, and in some cases exhibited further aggregation over time (Figure S4).

**Light Transmission as a function of Fe concentration**

Light transmission decreased with increasing Fe concentration in both sea and fresh water media (Figure S5). The decrease in transmission for Nanofer 25S and Nanofer STAR was much more exponential than for the bulk Fe^2+^ and Fe^3+^ in both sea and freshwater media. The decrease was less significant at concentrations below 50 mg/L, so the impact of this decrease in light on the growth of phytoplankton may have been minimal.

**Particle Charge**

Generally, the charge on the Nanofer 25 particles varied considerably and was closer to neutral than the charge on the Nanofer 25S particles, which was near -40 mV. Neutral particles tend to aggregate faster unless a bulky coating is added to the nZVI. Aggregation of the particles was high when the charge was small, below around ± mV. Thus, the zeta potential of the particles in a given media can be used to predict whether the particles will be stable or not (Table S1).

**pH of Fe in Media Over Time**

In both sea and fresh water media, the pH decreased by less than 4% over the four days of the phytoplankton experiment, with the exception of the Nanofer STAR and Nanofer 25S in fresh water media at some high concentrations where the pH actually increased by 2-3%. For the most part, the change in pH was larger at lower concentrations (Table S2).
